# The Challenge of Identifying Representative Samples in Research Involving Minority Participants

**DOI:** 10.1093/geroni/igab046.381

**Published:** 2021-12-17

**Authors:** Giovanna Pilonieta, David Geldmacher

**Affiliations:** 1 University of Alabama At Birmingham, Birmingham, Alabama, United States; 2 University of Alabama at Birmingham, University of Alabama at Birmingham, Alabama, United States

## Abstract

Determining participants' demographics, cognition, and functional performance by race is crucial to understanding disparities in clinical research on Alzheimer’s disease. We compared demographic and performance variables between Black/African American (B/AA; N=30; 41%) and White participants (N=43, 59%) in the UAB Alzheimer's Disease Center. Among 73 participants, 38 (52%) were women, mean age was 65.7 (SD 9.47), and mean education was 16 (2.31) years. Significant differences in gender proportions across race groups were observed. B/AA women represented 70 % of their race group, white women represented 39.5 %. There were no statistically significant differences in age, education, cognitive or functional severity, reasons to participate in research, referral source, objective measures of cognition, or informant-rated daily function by race group. In conclusion, despite 50% oversampling of B/AA participants compared to the State population, no differences in cognitive and functional performance at the time of enrollment were associated with race.

